# Supplementary material for: Population structure and transmission of Mycobacterium bovis in Ethiopia
Source: Microb Genom. 2021 May 4;7(5):000539. doi: 10.1099/mgen.0.000539 (PMC8209724; doi:10.1099/mgen.0.000539)
Supplement: Supplementary material 1 [file mgen-7-0539-s001.pdf]

Supplementary Table 1: Model performance based on marginal likelihood estimates (MLE) Bayes factors.

**Model**	**log MLE**	**log Bayes Factor**	**Strength of Evidence (Kass & Raftery, 1995)**
Relaxed constant	-5604954.493	-	-
Relaxed exponential	-5604956.354	1.861248229	Positive
Strict exponential	-5605103.905	149.4115816	Very strong
Strict constant	-5605106.444	151.9514509	Very strong

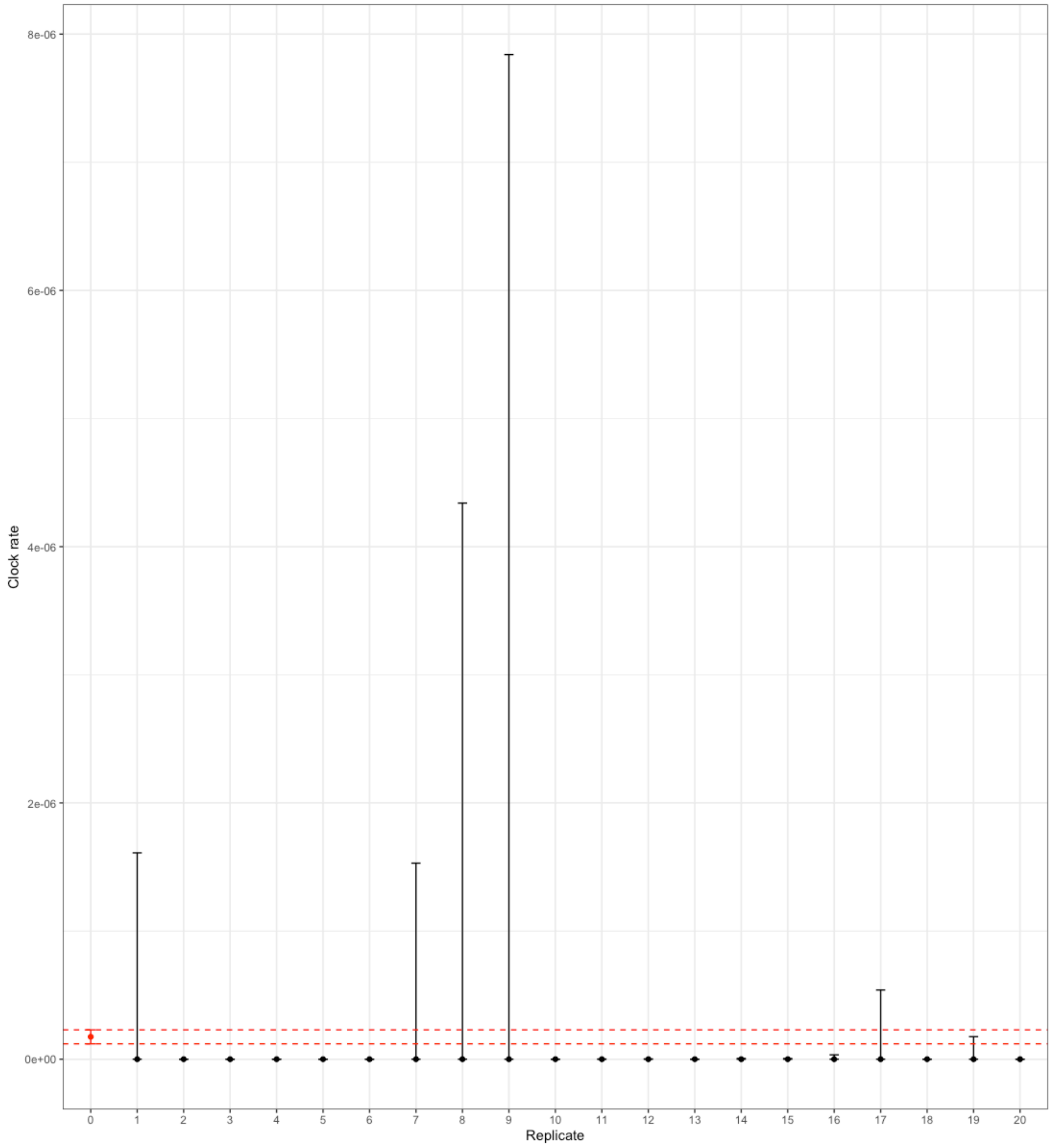

Supplementary Figure 1: BEAST dated tip randomization (DTR) analysis. Observed data is highlighted in red.
